# Acetylation of Chromatin-Associated Histone H3 Lysine 56 Inhibits the Development of Encysted *Artemia* Embryos

**DOI:** 10.1371/journal.pone.0068374

**Published:** 2013-06-19

**Authors:** Rong Zhou, Fan Yang, Dian-Fu Chen, Yu-Xia Sun, Jin-Shu Yang, Wei-Jun Yang

**Affiliations:** Key Laboratory of Conservation Biology for Endangered Wildlife of the Ministry of Education and College of Life Sciences, Zhejiang University, Hangzhou, Zhejiang, People’s Republic of China; Duke University, United States of America

## Abstract

**Background:**

As a response to harsh environments, the crustacean *artemia* produces diapause gastrula embryos (cysts), in which cell division and embryonic development are totally arrested. This dormant state can last for very long periods but be terminated by specific environmental stimuli. Thus, *artemia* is an ideal model organism in which to study cell cycle arrest and embryonic development.

**Principal Finding:**

Our study focuses on the roles of H3K56ac in the arrest of cell cycle and development during *artemia* diapause formation and termination. We found that the level of H3K56ac on chromatin increased during diapause formation, and decreased upon diapause termination, remaining basal level throughout subsequent embryonic development. In both HeLa cells and *artemia*, blocking the deacetylation with nicotinamide, a histone deacetylase inhibitor, increased the level of H3K56ac on chromatin and induced an artificial cell cycle arrest. Furthermore, we found that this arrest of the cell cycle and development was induced by H3K56ac and dephosphorylation of the checkpoint protein, retinoblastoma protein.

**Conclusions/Significance:**

These results have revealed the dynamic change in H3K56ac on chromatin during *artemia* diapause formation and termination. Thus, our findings provide insight into the regulation of cell division during arrest of artemia embryonic development and provide further insight into the functions of H3K56ac.

## Introduction

Dormancy is a period in an organism’s life cycle when growth, development, and physical activity are greatly slowed or even reversibly stopped, and these events tend to be closely associated with environmental conditions [1]. During dormancy, cell division is slowed or arrested, and metabolic activity is reduced, enabling the organism to conserve energy [2–4]. The primitive crustacean*, Artemia*, possesses two independent reproductive pathways that allow adaptation to different conditions. In one path motile nauplius larvae are released, generally under favourable surroundings. However, when encountering harsh environmental conditions, the other pathway is taken, in which cell division and development are arrested at the gastrulae stage [5]. These diapause cysts are in an obligate but reversible dormant state. Under certain environmental stimuli diapause is terminated, with the conversion of diapause cysts into post-diapause cysts. Under suitable conditions, post-diapause cysts develop into motile nauplius larvae [6]. Therefore, the life cycle of *Artemia* provides a very useful system in which to study the molecular mechanisms underlying the regulation of cell division and embryonic development. Much work has been done previously on the regulation of diapause in *Artemia* [7–10]. However, the role of epigenetic regulation in controlling the cell cycle and embryonic development associated with diapause remains unknown.

Histone post-translational modifications (PTMs), including acetylation, phosphorylation, methylation, ubiquitination, and SUMOylation, play important biological functions in diverse cellular processes [11]. The different effects elicited by each PTM and the various combinations of these PTMs are termed the “histone code” [12]. The conformation of chromatin is predominantly determined by histone acetylation, which is controlled by histone acetyltransferases (HATs) and histone deacetylases (HDACs) [13,14]. These modifications regulate nucleosome assembly, folding and compaction of chromatin which are essential for DNA replication and gene transcription [14].

Lysine 56 is the last residue of the αN-helix of H3 in the globular domain and located at the entry and exit points of the nucleosome core particle [15]. Thus, lysine 56 in histone H3 is a critical residue for various modifications. Recently, acetylation at this residue has been shown to occur in budding yeast and human cells [16,17]. Acetylation of histone H3 lysine 56 (H3K56ac) can weaken the binding between the histone octamer and DNA, and is therefore involved in a variety of processes [15,16].

Previous reports have shown that H3K56ac participates in cell cycle regulation both during normal replication-coupled nucleosome assembly, and also in response to DNA damage in yeast [15,18–21]. In the unperturbed cell cycle of yeast, newly synthesized histone H3 is first acetylated at lysine 56 by Rtt109, a process facilitated by the histone chaperone Asf1 [18,22]. Subsequently, Caf1 and Rtt106 mediate the deposition of histone octamers with H3K56ac onto chromatin during S phase [23]. After H3K56ac is removed from chromatin by Hst3 and Hst4, cells enter into G2 phase [24]. Thus, the dynamic equilibrium of H3K56ac, balanced by HAT and HDAC, plays an important role in the normal cell cycle progression. Yeast with mutations in HATs or HDACs exhibit an increased sensitivity to DNA damage [25,26]. The histone deacetylase responsible for H3K56ac has been reported to be sirtuins, a conserved family of nicotinamide adenine dinucleotide (NAD)^+^-dependent protein deacetylases, and its special inhibitor is nicotinamide (NM) [17,27]. In response to S phase-coupled genotoxic stress, accumulation of checkpoint-dependent H3K56ac, mediated by Hst3 proteolysis, was observed and resulted in a defect in the S phase DNA damage checkpoint [28]. Moreover, Tyler group reported that the persistence of H3K56ac on chromatin regulates chromatin disassembly and reassembly during DNA repair, and signals the completion of DNA repair necessary to satisfy the checkpoint and allow recovery of the cell cycle [29].

The present study focused on the role of H3K56ac in cell cycle arrest in the diapause embryo of *Artemia*. The results showed that the level of H3K56ac on chromatin was high in diapause embryos, reduced in post-diapause (activated) embryos, and remained basal level throughout the rest of post-diapause development. Furthermore, perturbation of HDAC function both in *Artemia* and HeLa cells led to an increase in H3K56ac on chromatin with subsequent cell cycle arrest. Taken together, our findings indicated that the dynamic change of H3K56ac on chromatin, regulated by HDAC activity, plays a critical role in cell cycle arrest in diapause embryos and their development.

## Materials and Methods

### *Animals and sample collecting*

*Artemiaparthenogenetica* was cultured at 25^º^C in a light-dark cycle of L:D=16:8 (4% seawater) and a light-dark cycle of L:D=5:19 (8% seawater) to release nauplius larvae and diapauses cysts respectively. Animals were fed with *Chlorella* powder every two days. The morphology of *Artemia* shell glands was used to differentiate between the two reproduction pathways [30]. Different developmental stages were identified as previously described [31,32].

To prepare post-diapause (activated) embryos, cysts directly released by adult *Artemia* were dehydrated in saturated sodium chloride solution for 24 h and then frozen at -20^º^C for three months. In hatching process, activated cysts were first rehydrated at 4^º^C for 5 h, and then incubated in 2% artificial seawater at 25 ^°^C with constant light. Samples were collected at 0, 4, 8, 12, 16, 24, 36h and 48h, respectively.

### *Inhibitor treatment*

As an inhibitor of sirtuins, nicotinamide (NM) (Sangon, Shanghai, China) was used to increase the level of H3K56ac. In NM treatment experiment, rehydrated post-diapause embryos were decapsulated in 3% sodium hypochlorite solution for 15 minutes and then were incubated in 2% seawater containing 40mM NM for 24h. After that the inhibitor was removed and the embryos were kept in 2% seawater for another 48h. Samples were collected every four hour. Decapsulated and rehydrated post-diapause embryos cultured constantly in 2% seawater were used as the control group.

### *Histone purification*

Histones were purified according to an acid extraction protocol [33]. In brief, tissues or cells were homogenized in hypotonic lysis buffer (10 mM Tris-Cl pH 8.0, 1 mM KCl, 1.5 mM MgCl_2_, 1 mM DTT and proteinase inhibitors). After centrifugation at 10000 *g* for 10 minutes, the pellet was resuspended in 0.4 N H_2_SO_4_ and the tubes rotated overnight at 4^º^C, and then TCA-acetone precipitation was performed. The purified histones were dissolved in 1× PBS buffer. Purified histones were examined by Coomassie staining, and then analysed by Western blotting with anti-H3, anti-H3K56ac and anti-H3S10p antibodies (Epitomics, Burlingame, CA, USA).

### *Generation of chromatin and non-chromatin fractions*

NP-40-based fractionation was performed as previously described [17,34]. Briefly, samples were lysed in the low salt NP-40 buffer (20 mM Tris-Cl pH 8.0, 150 mM NaCl, 1% NP-40, 1 mM DTT and proteinase inhibitors) and the lysate was centrifuged at 3300 *g* for 10 min at 4^º^C. The first supernatant (S1) was collected. The pellet was resuspended in the high salt NP-40 buffer (20 mM Tris-Cl pH 8.0, 450 mM NaCl, 1% NP-40, 1 mM DTT and proteinase inhibitors), placed on ice for 10 min, and then centrifuged at 13000 *g* for 10 min at 4^º^C. This second supernatant (S2) was collected. The S1 and S2 fractions were combined to generate the non-chromatin fraction. The pellet is the chromatin fraction. The protein compositions of chromatin and non-chromatin fractions were evaluated by Coomassie-stained gels.

### *BrdU incorporation assay*

*Artemia* were incubated in seawater containing 1 mM BrdU for 24h, fixed with 4% paraformaldehyde and paraffin-embedded. 6 µm-thick sections were incubated with a mouse monoclonal anti-BrdU antibody (Sigma-Aldrich, St. Louis, MO, USA) at 4^º^C overnight, and then with an anti-mouse IgG-AP-conjugated antibody (HuaAn Biotechnology, Hangzhou, China). Staining was performed using NBT/BCIP solution (Promega, Madison, WI, USA) in the dark. The staining reaction was stopped by the addition of 10 mM TE buffer (10mM Tris-Cl pH 8.0, 1mM EDTA).

### *Cell culture, transfection and flow cytometry*

HeLa cells were cultured in Dulbecco’s Modified Eagle’s medium (Gibco, Langley, OK, USA) supplemented with 10% FBS (Gibco, Langley, OK, USA), and grown in 5% CO_2_ at 37^º^C. Cells were treated with 25 mM NM (BBI, Milford, CT, USA) for 24 h. The sequences of siRNAs targeting the human genes encoding Sirt1 (accession #NM_001142498.1) and Sirt2(accession #NM_012237.3) have been previously reported [17]. Sirt1 siRNA: 5'-ACUUUGCUGUAACCCUGUA(dTdT)-3', Sirt2 siRNA: 5'-GACUCCAAGAAGGCCUACA(dTdT)-3'. Control siRNA targeted the human gene encoding luciferase. siRNAs were synthesized using the *in vitro* transcription T7 Kit (Takara Bio, Shiga, Japan) in accordance with the manufacturer’s instructions. Cell transfection was performed using Lipofectamine 2000 (Invitrogen, Carlsbad, CA, USA) transfection reagent according to the manufacturer’s standard protocol. The cells with siRNA or NM treatment were harvested with trypsin and fixed with 70% ethanol overnight at 4^º^C. Fixed cells were treated with RNase A (BBI, Milford, CT, USA) for 30 min at 37^º^C and stained with propidium iodide (BBI, Milford, CT, USA) for 30 min at 4^º^C in the dark. The percentage of cells in each cell cycle phase was measured using a flow cytometer (Beckman Coulter, FC500MCL).

### *Molecular cloning of Rtt109 and Asf1*

Total RNA was extracted from nauplius larvae using the TRIzol Reagent (Invitrogen, Carlsbad, CA, USA) according to the manufacturer’s instructions. First-strand cDNA was synthesized from 1 µg of total RNA by M-MLV Reverse Transcriptase (Takara Bio, Shiga, Japan) in a 10-µl reaction. According to the known sequences of Rtt109 and Asf1 in other species, two pairs of generative primers (ArRTT109-F3 and ArRTT109-R3, ArASF1-F1 and ArASF1-R2; Table 1) were designed and a two-round PCR amplification was performed. Amplified fragments were subcloned into the vector pUCm-T (Sangon, Shanghai, China) and sequenced with M13F/R. Both the sequenced cDNA and the deduced peptide were analyzed by EditSeq v 5.00 (DNAStar, Madison, WI, USA), and Blast was performed using the NCBI website to confirm their homologies. The partial nucleotide sequences of Rtt109 and Asf1 encoding cDNAs were submitted to GenBank and the accession numbers were KF030132 and KF030133.

**Table 1:** Nucleotide sequences of primers used in polymerase chain reactions.

Primer	Length(bp)	Direction	Sequence(5’–3’)
ArASF1-F1	20	F	GTNGTNGTNYTNGAYAAYCC
ArASF1-R2	20	R	GGNGGRTTYTCNCKNARYTC
ArRtt109-F3	20	F	TNGARGTNAARCCNGGNATG
ArRtt109-R3	20	R	AARTCNCCYTCRAARTANGG
ArASF1-RTF2	26	F	GATAATCCATCAATGTTCTCCAGCCC
ArASF1-RTR2	29	R	ACGTAATAGCCAACACGTAAAAACTCTTG
ArRtt109-RTF2	26	F	GTTCAGACTCGCCAATGCCAAATACG
ArRtt109-RTR2	24	R	TCATCACCTTCGGACGGAGGACAA
Tubulin-RTF	20	F	GCAGTGGTCTACAAGGTTTC
Tubulin-RTR	22	R	ATCAAAACGAAGGCTGGCGGTG

F and R indicate the forward and reverse directions, respectively

### *Virtual Northern blotting*

Virtual Northern blotting is a method to verify differential gene transcription. This method, which requires only minute amounts of RNA, was used as an alternative to conventional Northern blotting [35]. Rtt109 and Asf1 ortholog fragments were amplified by using the primer combinations ArRtt109-RTF2/ArRtt109-RTR2 and ArASF1-RTF2/ArASF1-RTR2, respectively, in 25-µl reactions using 0.5 µl of reverse transcription product of each stage as the template. As an internal control, a fragment of tubulin was amplified with the primers Tubulin-RTF and Tubulin-RTR. 200 ng of each purified PCR fragments (Rtt109, Asf1 and tubulin) were labelled in a 5-µl reaction with DIG High Prime DNA Labelling Kit (Roche, Mannheim, Germany) at 37^°^C overnight to prepare DIG-labelled probes. To obtain semi-quantitative mRNA levels, a non-saturating number of cycles were used (12, 12 and 6 cycles for Rtt109, Asf1 and tubulin respectively). 10 µl of aliquots of PCR products were fractionated on 1.5% agarose gel and transferred onto a positively-charged nylon membrane (Millipore, Bedford, MA, USA). After pre-hybridization at 42^°^C for 1 h, the membrane was hybridized at 42^°^C overnight with DIG-labeled probes. After extensive washing, hybridized probes were visualized using a DIG chemiluminescent detection system (Roche, Mannheim, Germany).

All amplifications were done on a TGradient thermocycler (Whatman-Biometra, Göttingen, Germany) and all sequencings were performed on ABI 3730 automated sequencer (Applied Biosystems, Foster City, CA, USA).

### *Western blotting*

Protein extraction was performed with TRIzol (Invitrogen, Carlsbad, CA, USA). Whole protein extract (40 µg) and purified histones (5 µg) were separated on 10% or 12.5% SDS-PAGE gels, respectively, and transferred to PVDF membranes (Roche, Mannheim, Germany), which were immunoblotted with primary antibodies at 4^º^C overnight. Blocking buffer, secondary antibodies, and chemiluminescence solution were provided in the BM Chemiluminescence Western Blotting Kit (Roche, Mannheim, Germany), and all steps were performed according to the manufacturer’s standard protocol. The following primary antibodies were used: anti-histone H3, anti-H3K56ac, anti-H3S10p, anti-CDK4, anti-CDK6, anti-phospho-RbT356 (Epitomics, Burlingame, CA, USA), anti-phospho-RbS807/811 (Cell Signaling Technology, Danvers, MA, USA), anti-Rb (Santa Cruz Biotechnology, Santa Cruz, CA, USA), anti-Tubulin (Sigma-Aldrich, St. Louis, MO, USA).

Bands on blots were quantified by measuring the intensities using ImageJ. These intensities were normalized against those of the loading controls, tubulin and histone H3. The ratio of H3K56ac in the pellet relative to that in the supernatant was quantified by the normalized intensities and described in histograms. Statistical significance was determined using the Student’s *t*-test.

## Results

### *Cell cycle and developmental arrest in Artemia diapause embryos*

**Figure 1:**
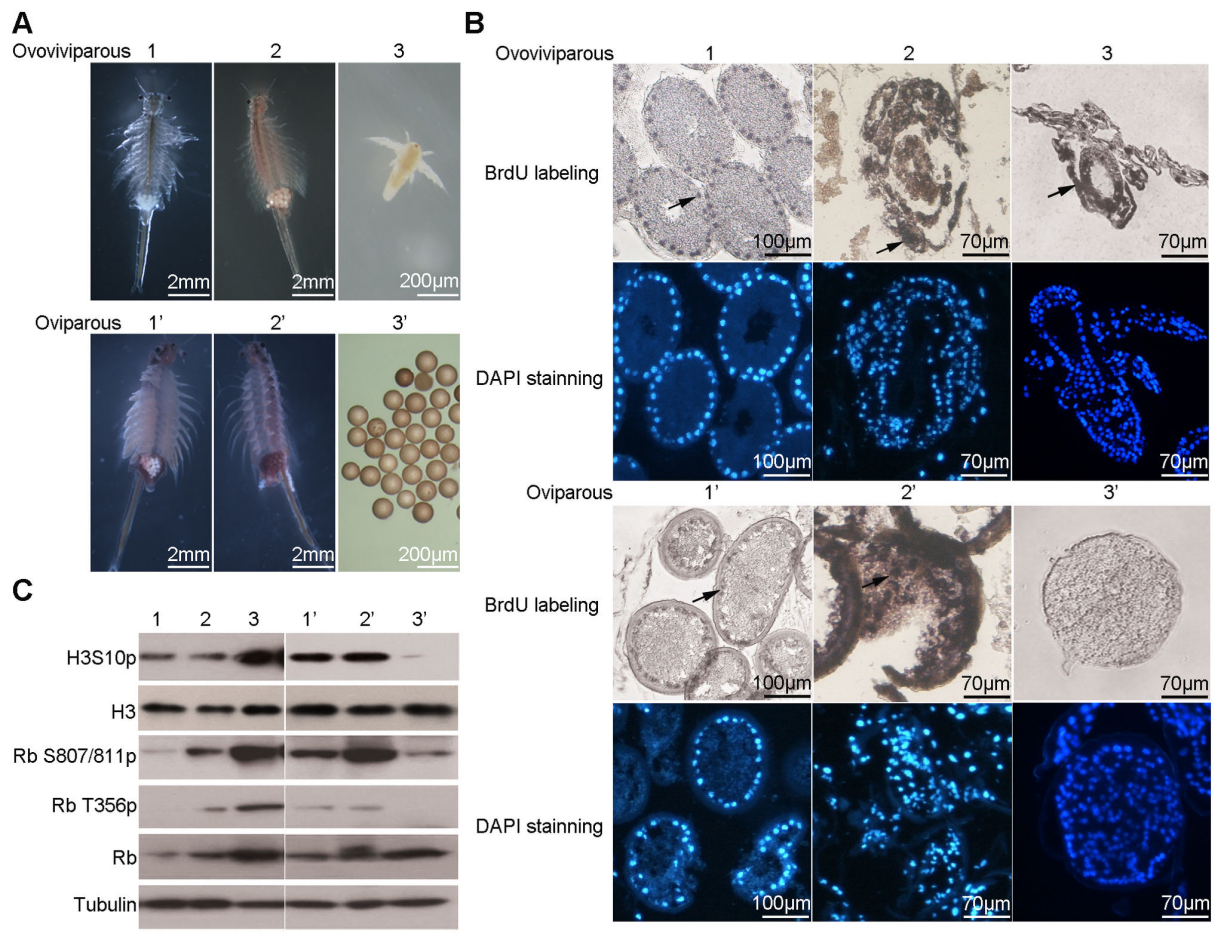
Cell cycle arrest in *Artemia* diapause embryos. 1–3 and 1’–3’ represent three embryonic developmental stages in ovoviviparous and oviparous pathway, respectively. 1 and 1’, early embryo (two days after the eggs entering into the ovisac); 2 and 2’, late embryo (four days after the eggs entering into the ovisac); 3, nauplius; 3’, diapause embryo. (**A**) Morphology of *Artemia* adults with embryos in the ovisac. (**B**) BrdU incorporation assay (upper panel) and corresponding DAPI staining (lower panel) with embryos or nauplius of the two reproduction pathways. Black arrows indicated the representative positive signal. (**C**) Western blotting analysis of cell division-related molecules at each stage of ovoviviparous and oviparous reproduction pathways. Tubulin was used as a loading control for the whole protein extracts, and H3 used as a loading control for the total histones.

*Artemia* has two modes of reproduction: ovoviviparous and oviparous. In the ovoviviparous pathway, oocytes formed in the ovary and matured in the oviduct in the early and late oocyte stages, respectively. Embryos then entered the ovisac (uterus) and finally released into the environment as nauplius larvae. In the oviparous pathway, embryos in the ovisac are covered with a chitinous shell, and released into the environment as encysted diapause embryos. To compare these two reproductive modes, the stages of embryonic development of each pathway were studied (Figure 1A).

A BrdU incorporation assay was used to characterize the cell cycle arrest in diapause embryos. Cell division was observed in early and late embryos after oocytes entered the ovisac in both reproduction modes. However, the cells of motile nauplius larvae continued to divide, while the cells of diapause embryos were arrested (Figure 1B).

This difference in cell division between nauplius larvae and diapause embryos was confirmed by Western blot analysis of histone H3 phosphorylated at serine 10 (H3S10) and phosphorylated retinoblastoma protein [36]. High levels of phosphorylated H3S10 and Rb were detected in nauplius larvae, reflecting the high level of cell division there. In contrast, the levels of phosphorylated H3S10 and Rb were lower in diapause embryos, indicating that the cell cycles had been arrested at this stage (Figure 1C).

### *Accumulation of H3K56ac on chromatin in Artemia diapause embryos*

**Figure 2:**
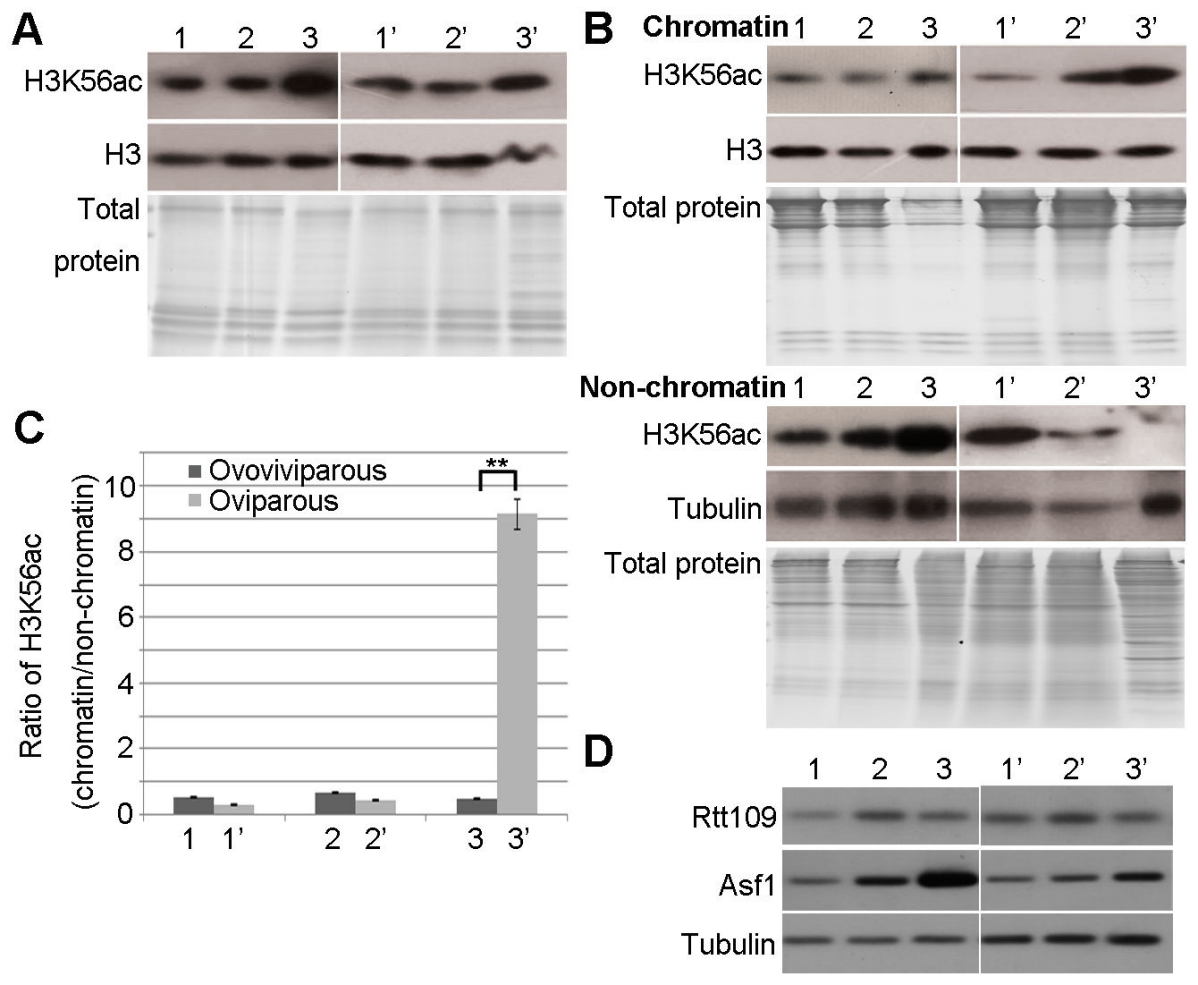
Accumulation of H3K56ac on chromatin in *Artemia* diapause embryos. (**A**) The total level of H3K56ac at each developmental stage of the ovoviviparous and oviparous pathways was examined by Western blotting. The purity of histones was evaluated with a Coomassie-stained gel. The developmental stages are labelled 1–3 and 1’–3’, as described in Figure 1. Histone H3 was used as a loading control. (**B**) Western blotting for H3K56ac in the chromatin and non-chromatin fractions of samples at stages 1–3 and 1’–3’. Coomassie-stained gels showed the protein composition of chromatin and non-chromatin fractions. Tubulin was used as a loading control for non-chromatin fractions. Histone H3 was used as a loading control for chromatin fractions. (**C**) The intensities of the H3K56ac ECL signals were measured, and the ratio of H3K56ac in the chromatin fraction relative to H3K56ac in the non-chromatin fraction was calculated (chromatin/non-chromatin) and is shown in the bar graph. The means of three independent biological replicates are shown; error bars represent the S.E.M. The difference in chromatin/non-chromatin between nauplius larvae and diapause embryos was evaluated using a Student’s *t*-test. ** indicates P<0.01. (**D**) Expression of Rtt109 and Asf1 during the different reproduction pathways examined by virtual Northern blotting.

To explore the relationship between the total level of H3K56ac and the formation of diapause embryos, purified histones were analysed by Western blotting. The total level of H3K56ac was similarly high at all developmental stages and in both developmental modes (Figure 2A). To understand the similar level of H3K56ac in the two modes, we fractionated whole cell extracts to obtain chromatin and non-chromatin fractions. Western blot analysis indicated that the level of H3K56ac on chromatin was much higher, while the level of H3K56ac in the non-chromatin fraction was relatively lower in diapause embryos than in nauplius larvae (Figure 2B). Therefore, the ratio of H3K56ac on chromatin relative to that in the non-chromatin fraction was significantly different between the two pathways (Figure 2C). H3K56ac was mainly distributed in the non-chromatin fraction of nauplius larvae, whereas it was mainly bound to chromatin in diapause embryos (Figure 2B). These results suggest that the accumulation of H3K56ac on chromatin could play a critical role in cell cycle arrest in *Artemia* diapause embryos.

Rtt109 is a histone acetyltransferase in yeast, and Asf1 functions as the chaperone for Rtt109. These two proteins are reported to be important regulators of H3K56ac [18,22]. The alignments of amino acid sequences indicated that the *Rtt109* and *Asf1* orthologs are conserved in *Artemia* (Figure S1). We investigated the gene transcription of the *Rtt109* and *Asf1* orthologs in the two reproductive pathways of *Artemia* via virtual Northern blotting. The results indicated that the gene transcription of the *Rtt109* ortholog showed no significant difference between the different reproductive pathways. However, the gene transcription of *Asf1* ortholog was relatively high in the nauplius larvae, while lower in the diapause cysts (Figure 2D). These results indicated that Asf1 ortholog may regulate the levels of H3K56ac in the non-chromatin fraction in the diapause cysts and nauplius larvae.

### *Characterization of H3K56ac on chromatin during diapause termination in Artemia*

**Figure 3:**
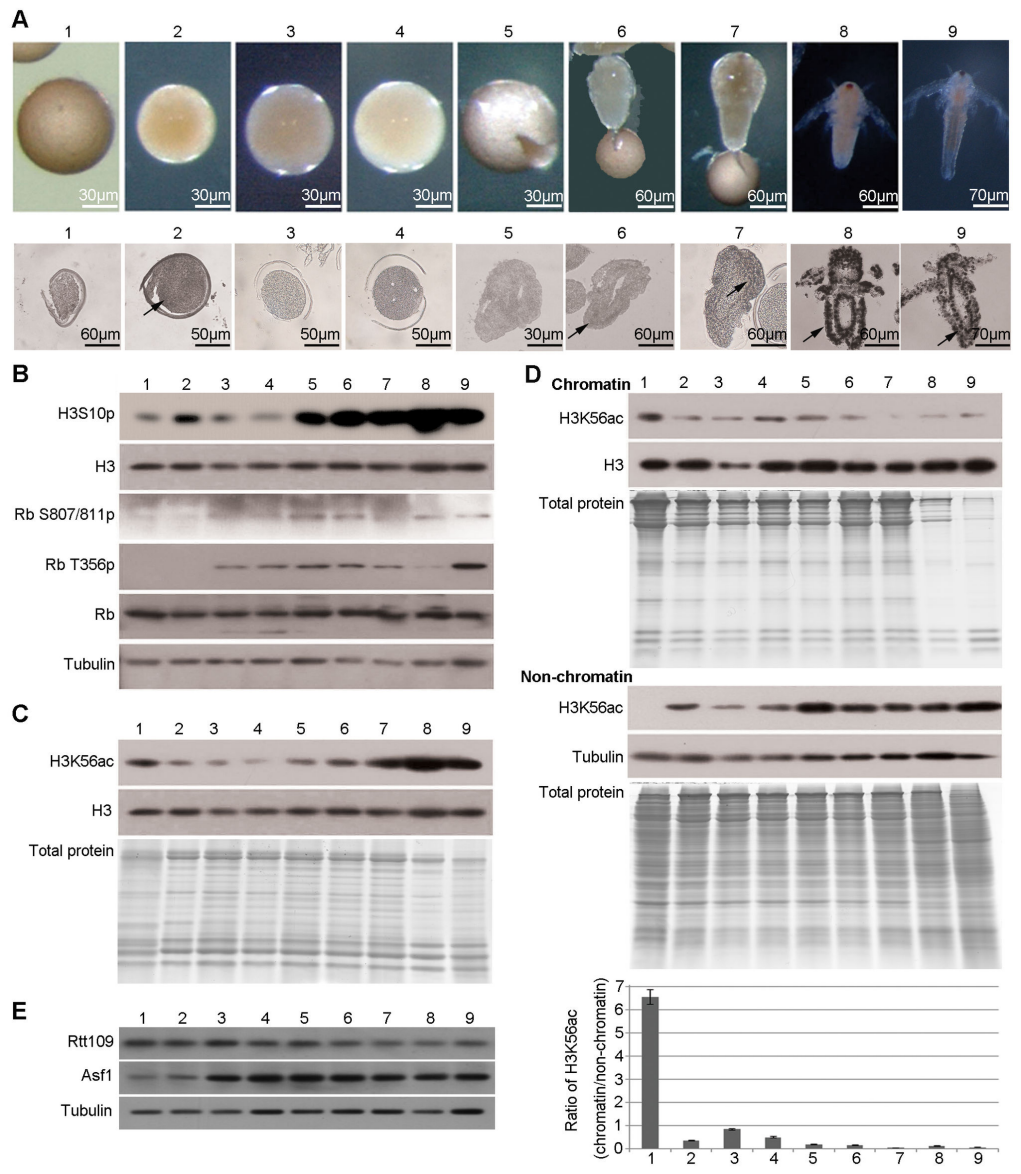
The amount of H3K56ac bound to chromatin decreases during diapause termination and post-diapause development. Diapause embryos, post-diapause embryos and samples during the hatching process were collected as mentioned above. (**A**) Morphology (upper panel) and BrdU incorporation assay (lower panel) of embryos and nauplius larvae at various developmental stages. The black arrows indicate the representative positive signal. (**B**) Western blotting analysis. Tubulin was used as a loading control. (**C**) Western blotting for H3K56ac in total histone extracts. The purity of histone extracts was evaluated by a Coomassie-stained gel. Histone H3 was used as a loading control. (**D**) Western blotting for H3K56ac in the chromatin and non-chromatin fractions. Coomassie-stained gels showed the protein composition of chromatin and non-chromatin fractions. Tubulin was used as a loading control for non-chromatin fractions. Histone H3 was used as a loading control for chromatin fractions. The intensities of the ECL signals were measured, and the ratio of H3K56ac in the chromatin fraction relative to that in the non-chromatin fraction was calculated (chromatin/non-chromatin) and is shown in the bar graph. The means of three independent biological replicates are shown; error bars represent the S.E.M. (**E**) Expression levels of Rtt109 and Asf1 by virtual Northern blotting.

Diapause was terminated as described, converting diapause embryos into post-diapause (activated) ones, but no morphological changes were observed during or after this transition. Pre-nauplius emerged from the cyst shell in embryos incubated for 16 to 20 h as described in Materials and Methods. The development of pre-nauplius was completed during the early nauplius stage. The morphological changes at each developmental stage during diapause termination and post-diapause development were observed (Figure 3A).

The BrdU incorporation assay was used to investigate cell cycle progression during diapause termination and post-diapause development. This result indicated that cells in post-diapause embryos passed through the cell cycle arrest state following diapause termination (Figure 3A). Consistent with previous studies [9], cell division did not occur during the pre-emergence stage. Mitogenesis began at the emergence stage and greatly increased in nauplius larvae (Figure 3A).

This result was validated by analysis phosphorylations of H3S10 and Rb during diapause termination and also during post-diapause development. H3S10 phosphorylation was increased a little during diapause termination, and then largely occurred from the emergence stage coupled with the onset of mitogenesis (Figure 3B). Rb was dephosphorylated during diapause termination and phosphorylated throughout the entire post-diapause development (Figure 3B).

We next investigated the relationship between H3K56ac and the resumption of the cell cycle and embryonic development. Total H3K56ac levels declined in post-diapause embryos, remained low in the pre-emergence stage, and increased during the emergence and nauplius stages (Figure 3C). Next, the H3K56ac levels in the non-chromatin fraction and on chromatin were examined during diapause termination and post-diapause development. The level of H3K56ac bound to chromatin decreased following diapause termination and remained basal level during post-diapause development (Figure 3D). However, the level of H3K56ac in the non-chromatin fraction was low in diapause embryos but increased upon the resumption of embryonic development (Figure 3D). In summary, we propose that the H3K56ac on chromatin is important in diapause maintenance and termination.

Gene transcription of the *Rtt109* and *Asf1* orthologs in diapause termination of *Artemia* was also investigated. The results showed that the gene transcription of *Rtt109* ortholog was constant, while gene transcription of the *Asf1* ortholog increased during the post-diapause developmental process (4h-48h) (Figure 3E). Therefore, we propose that Asf1 may regulate H3K56ac levels in the non-chromatin fractions, and important for resumption of the cell cycle and embryonic development following diapause termination.

### *H3K56ac on chromatin regulates the cell cycle in HeLa cells*

**Figure 4:**
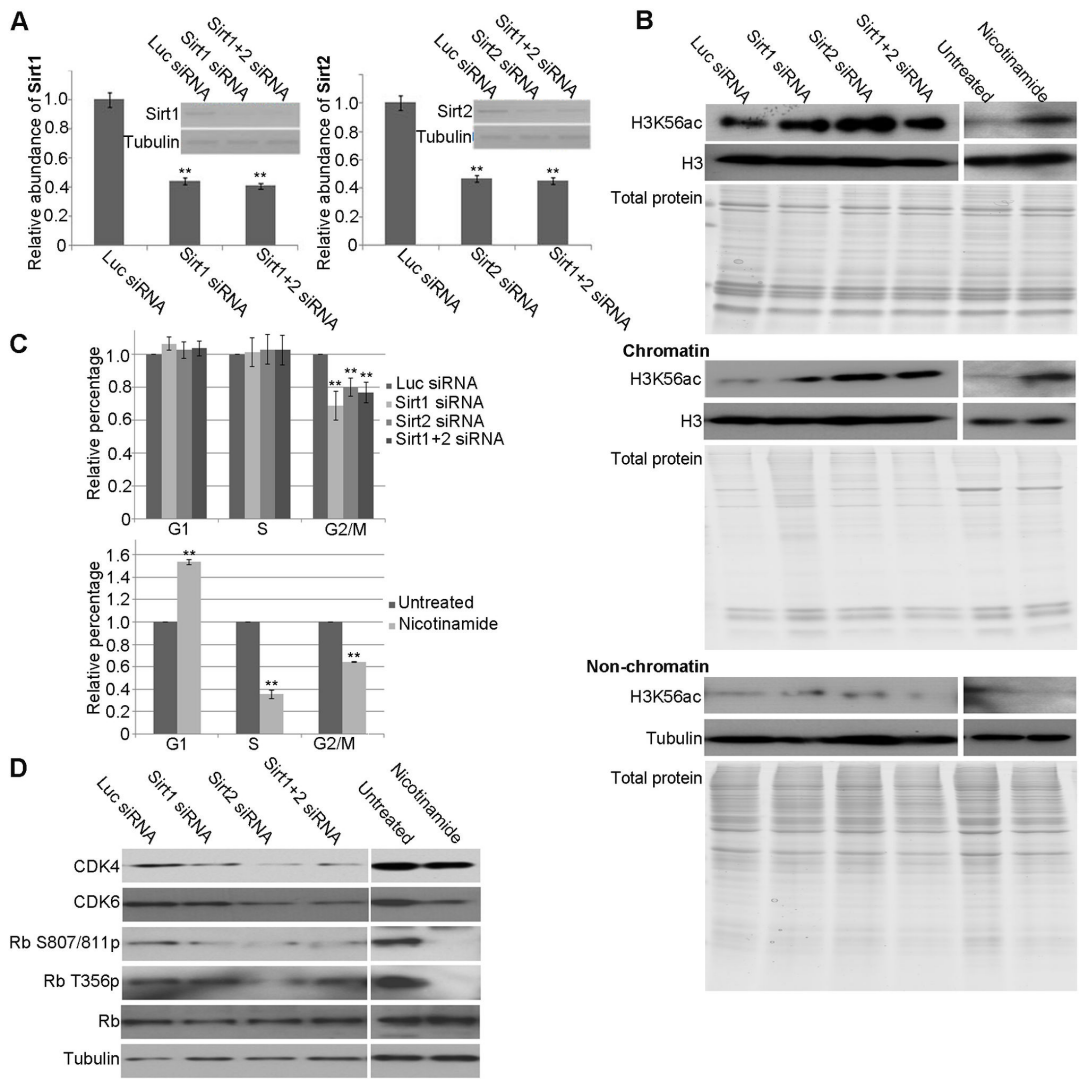
Pertubation of HDAC activity disrupts the cell cycle in HeLa cells. (**A**) The efficiency of *Sirt1* and *Sirt2* depletion in HeLa cells following treatment with siRNAs targeting *Sirt1* and/or *Sirt2* was evaluated by semi-quantitative PCR. Luciferase siRNA was used as a negative control. Tubulin was used as a loading control. (**B**) Western blot analysis of H3K56ac in total histone extracts, chromatin, and non-chromatin fractions of HeLa cells after the indicated treatments. The purity of histone extracts and protein composition of chromatin and non-chromatin fractions were evaluated by Coomassie-stained gels. Histone H3 was used as a loading control for the total histone extract and chromatin fractions. Tubulin was used as a loading control for the non-chromatin fractions. (**C**) The bar graphs show the relative percentage of cells in G1, S, and G2/M phase analysed by flow cytometry after various treatments. (**D**) Western blotting of total HeLa cell extracts following the indicated treatments. Tubulin was used as a loading control. In all cases, the means of three independent biological replicates are shown; error bars represent S.E.M Significant differences were evaluated using a Student’s *t*-test. ** indicates P<0.01.

To understand the relationship between the level of H3K56ac on chromatin and cell cycle arrest, we used HeLa cells as a model, which eliminated the influence of embryonic development. Previous studies indicated that Sirt1 and Sirt2 are the main HDACs responsible for the deacetylation of H3K56 in human cells *in vivo* [17]. In our study, RNA interference (RNAi) of *Sirt1 and/or Sirt2* and NM (a HDAC inhibitor) treatment were used to block deacetylation and increase the level of H3K56ac on chromatin. After transfection of siRNAs trargeting *Sirt1* and/or *Sirt2*, the mRNA level was less than 50% to the control (Figure 4A).

In contrast to the control, the level of total H3K56ac was higher in cells after RNAi of *Sirt1, 2* and both. Moreover, the level of H3K56ac on chromatin was increased in cells treated by RNAi compared with those in the controls (Figure 4B). Similarly, the levels of total and chromatin-bound H3K56ac were much higher in cells treated with NM than in control cells (Figure 4B). Flow cytometry was performed on these samples to determine their cell cycle phase (Figure S2). The ratio of cells in G2 phase was markedly lower in cells treated with *Sirt1*, *2* and both or NM than in control cells, whereas the number of cells in G1/S was more (Figure 4C). The results suggested that the accumulation of H3K56ac on chromatin leads to G1/S cell cycle arrest.

The expression of CDK4, CDK6, and the phosphorylation of Rb were examined by Western blotting to clarify the cell cycle phase of cells following the RNAi and NM treatments. The expression of CDK4 was decreased in *Sirt1*, *2* and both RNAi-treated cells, while the expression of CDK6 was decreased in *Sirt2* and both RNAi-treated cells. In addition, CDK6 was decreased, but CDK4 did no change in cells treated with NM (Figure 4D). Moreover, the levels of phosphorylated Rb were lower in *Sirt1, 2* and both RNAi-treated or NM treated cells (Figure 4D). Taken together, these results indicate that the accumulation of H3K56ac on chromatin, due to repressed deacetylation activity, activates the cell cycle checkpoint and results in G1/S cell cycle arrest.

### *In vivo repression of HDAC activity induces artificial cell cycle and developmental arrest in Artemia*

**Figure 5:**
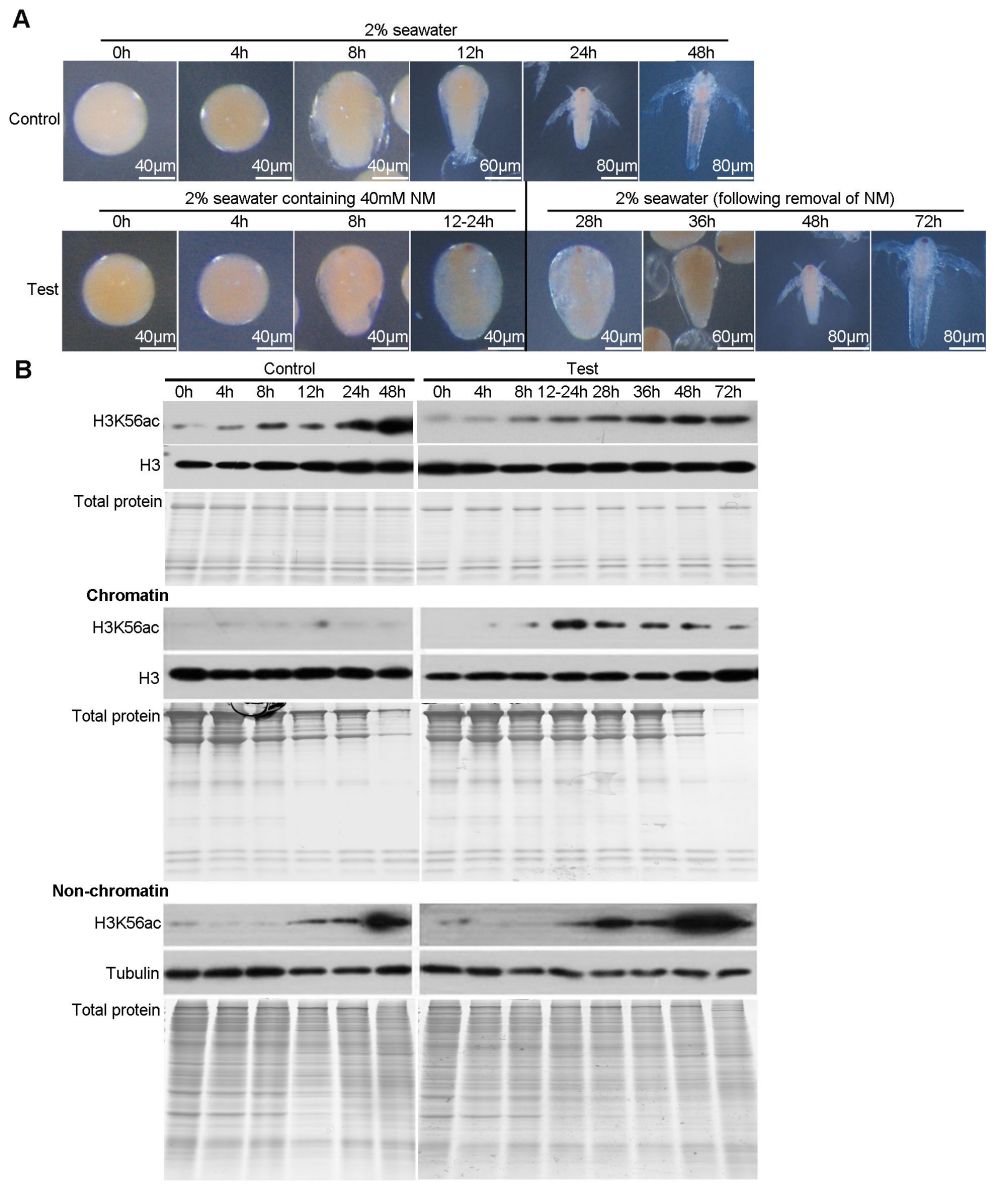
NM induces an artificially arrest via increasing the H3K56ac on chromatin during post-diapause development. (**A**) Morphology of embryos in control group (all 48h in 2% seawater) and test group (0h-24h in 2% seawater with NM and the following 48h in 2% seawater without NM) (**B**) Western blotting for H3K56ac in total histone extracts, chromatin, and non-chromatin fractions of control and test samples from the indicated time points. The purity of histone extracts and protein composition of chromatin and non-chromatin fractions were evaluated by Coomassie-stained gels. Tubulin was used as a loading control for non-chromatin fractions. Histone H3 was used as a loading control for chromatin fractions and total histone extracts.

According to the results above, we suggested that H3K56ac on chromatin plays a critical role in controlling the cell cycle arrest. Here, we treated the decapsulated post-diapause embryos with NM to block deacetylation. The morphologies of embryos treated with NM (0h-8h in test) were similar to those of controls (0h-8h in control) during the first 8 hours of embryonic development. However, in contrast to the controls (12h-48h in control), the embryonic development of NM treated embryos (12-24h in test) arrested at the pre-emergence stage which is just before cell division resumes (Figure 5A). Strikingly, these embryos developed into normal nauplius larvae when NM was removed (28h-72h in test) (Figure 5A).

The effect of NM on the level of H3K56ac during the post-diapause development was evaluated by Western blotting. The results revealed that the level of H3K56ac on chromatin increased in the embryos (12-24h in test) after NM treatment that the development arrested. However, the level gradually decreased to basal level after NM removal (Figure 5B). However, The level of H3K56ac in the non-chromatin fraction during the development was similar in the control and NM-treated embryos (Figure 5B). Therefore, NM increases the level of H3K56ac on chromatin by blocking deacetylation but does not affect acetylation activity.

**Figure 6:**
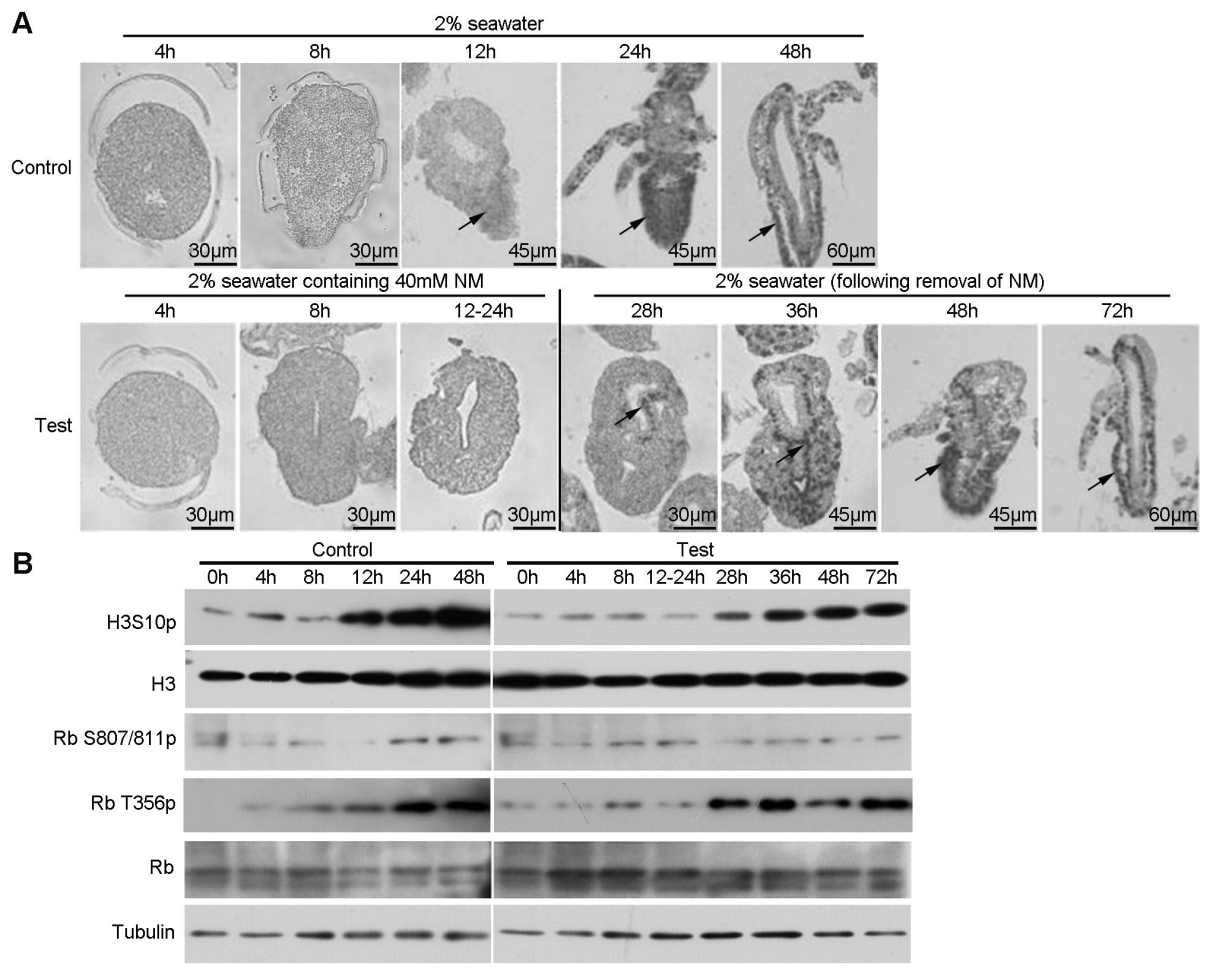
NM artificially arrests the cell cycle and development in post-diapause embryos. (**A**) BrdU incorporation assay of control and test groups in the NM treatment. Samples were treated and collected as described above. The black arrows indicate the representative positive signal. (**B**) Western blotting analysis. Tubulin and H3 were used as loading controls.

The BrdU incorporation assay and phosphorylation of H3S10 were used to indicate cell division during embryonic development. Cell division resumed at the emergence stage (12h in control), whereas did not occur in the arrested embryos (12-24h in test) after NM treatment (Figure 6A and 6B). Cell division resumed after NM is removed and the embryos developed into normal napulius larvae.

In general the cell cycle must be tightly regulated and this is usually dependent on checkpoint proteins, Rb [37]. Rb phosphorylation at Thr356 increased after the pre-emergence stage and reached high levels in nauplius larvae, but the phosphorylation of Ser807/811 had no change. Compared with that in control, phosphorylated Rb was maintained at a low level under NM conditions and increased after NM removal (Figure 6B). Based on these results, we hypothesize that high level of H3K56ac on chromatin induced the arrest of the cell cycle and embryonic development, mediated by dephosphorylation of Rb.

## Discussion

*Artemia* released diapause embryos under harsh environmental conditions. As mentioned here previously diapause can be terminated by certain conditions and the now “activated” embryos can developed into nauplius larvae when conditions became suitable [5]. According to previous reports and the present study, we can summarize the nature of cell division and embryonic development during these periods. Cell division and embryonic development take place to form the diapause (gastrula) embryos that are released to the aqueous environment, however, all cell division is arrest in diapause and post-diapause (activated) embryos. Following the diapause termination, embryonic development is resumed but cell cycle arrest continues until the emergence stage is reached and then cell division continues actively in the nauplius larvae [9]. In this study, H3S10p, a marker of M phase [38], increased dramatically from 12h, which indicates that mitogenesis began at this time. However, in our previous study, DAPI staining also showed that the mitogenesis began at the emergence stage from 12h but the rate of dividing cells was extremely low to under 1% [9]. The same phenomenon has also been reported by Olson and Clegg that development at the emergence stage only resulted in a 10% increase in nuclei numbers [39]. Although the mitogenesis began at 12h, the signal of BrdU labeling assay was weak due to small amounts of dividing cells. Many studies have been done to understand the basic mechanisms underlying *Artemia* diapause. For example, the molecular chaperones, p26 and artemin, are synthesized in great abundance and are specific to the diapause pathway where they play important roles in the integrity of proteins and RNA [7,8]. Kinases related to cell division, such as polo-like kinase 1 (Plk1) and ribosomal S6 kinase (RSK), are also involved in diapause formation (the former) and in the resumption of the cell cycle during post-diapause development (the latter) [9,10]. In addition, the energy sensor AMPK was used to evaluate metabolic activity, and found to be essentially undetectable [40]. However, in spite of these interesting results few studies have examined the epigenetic influence on *Artemia* diapause. As a result the current study was focused on the role of H3K56ac in the arrest of the cell cycle during *Artemia* diapause formation and termination.

H3K56ac is regulated in a cell cycle-dependent manner [15]. In the present study, significant amounts of H3K56ac were found on chromatin, while little was found in the soluble fraction of diapause embryos, in which cell division is arrested. This is consistent with previous studies showing that the persistence of H3K56ac on chromatin activates a cell cycle checkpoint [29]. However, studies on yeast revealed that the hyperacetylation on H3K56 perturbs replisomes and causes DNA damage [26]. In contrast, *Artemia* diapause cysts maintain a prolonged cell cycle arrest without loss of embryonic viability [5]. Thus, the genome stability of *Artemia* diapause cysts may be safeguarded by molecular chaperones, such as p26 [7], and by the complete cessation of DNA replication [5]. During the conservation of diapause cysts into the post-diapause state, H3K56ac on chromatin decreased. Previous studies reported that removal of H3K56ac from chromatin allows the cell cycle to enter the next phase and checkpoint recovery. Combined with the BrdU assay results, we suggested that the decreased H3K56ac on chromatin allowed the cell cycle to pass through S phase, providing for the resumption of cell division.

Acetylation is a dynamic process enabling regulation at the level of both acetylation and deacetylation [13]. Previous reports have indicated that Rtt109 is the main acetyltransferase functioning on H3K56, with Asf1 acting as the chaperone for Rtt109 [22]. Both proteins are important for the acetylation process. In the present study, we found that the level of H3K56ac in the non-chromatin fraction was low in diapause embryos, increased in post-diapause embryos, and was high in nauplius larvae. The mRNA expression level of the *Asf1* ortholog increased in a manner similar to that of the level of H3K56ac in the non-chromatin fraction during development. Thus, Asf1 was considered to regulate H3K56ac in *Artemia* for diapause maintenance and termination, but further study is necessary.

According to previous reports, three types of HATs have been isolated from *Artemia* nauplius larvae [41]. Acetylation activity gradually increased during post-diapause development [42]. Even though the *Rtt109* ortholog expressed at a constant level, a role for Rtt109 in acetylation cannot be excluded. That is because its activity might not be correlated with mRNA expression but rather dependent on the combination of activators. For example, polyamines have been reported to activate the acetylation of endogenous histones [43].

It was reported that, in both yeast and human cells, the deacetylase responsible for removing the acetyl group from H3K56 is the NAD-dependent HDAC, Sirtuins [17,44]. Studies in yeast indicated that the proteolysis of Hst3 would result in the accumulation of H3K56ac on chromatin [28]. In our study, increased acetylation of H3K56 on chromatin has been observed after blocking deacetylation with siRNAs or use of inhibitor (NM) both in HeLa cells and *Artemia*. These results suggest that HDAC activity is the main regulator of H3K56ac on chromatin in *Artemia* diapause embryos.

In the present study, siRNA interference and NM-treatment of HeLa cells revealed that increased H3K56ac on chromatin could result in cell cycle arrest. Moreover, both arrest embryos in the diapause and the NM-treated exhibited the accumulation of H3K56ac on chromatin as well as developmental arrest. In addition, cell division in the two kinds of embryos is restricted. The role of H3K56ac on the transcription network in human embryonic stem cells (ESCs) has also been reported and involved in maintaining the pluripotency [45]. The embryonic development with NM treatment was arrested at the pre-emergence stage before active mitogenesis. However, cell differentiation to form the eyespot and digestion cavity has indeed taken place. Thus, we speculate that the accumulation of H3K56ac on chromatin regulated embryonic development through controlling the cell cycle progression, but had no influence on cell differentiation based on the morphological evidence.

**Figure 7:**
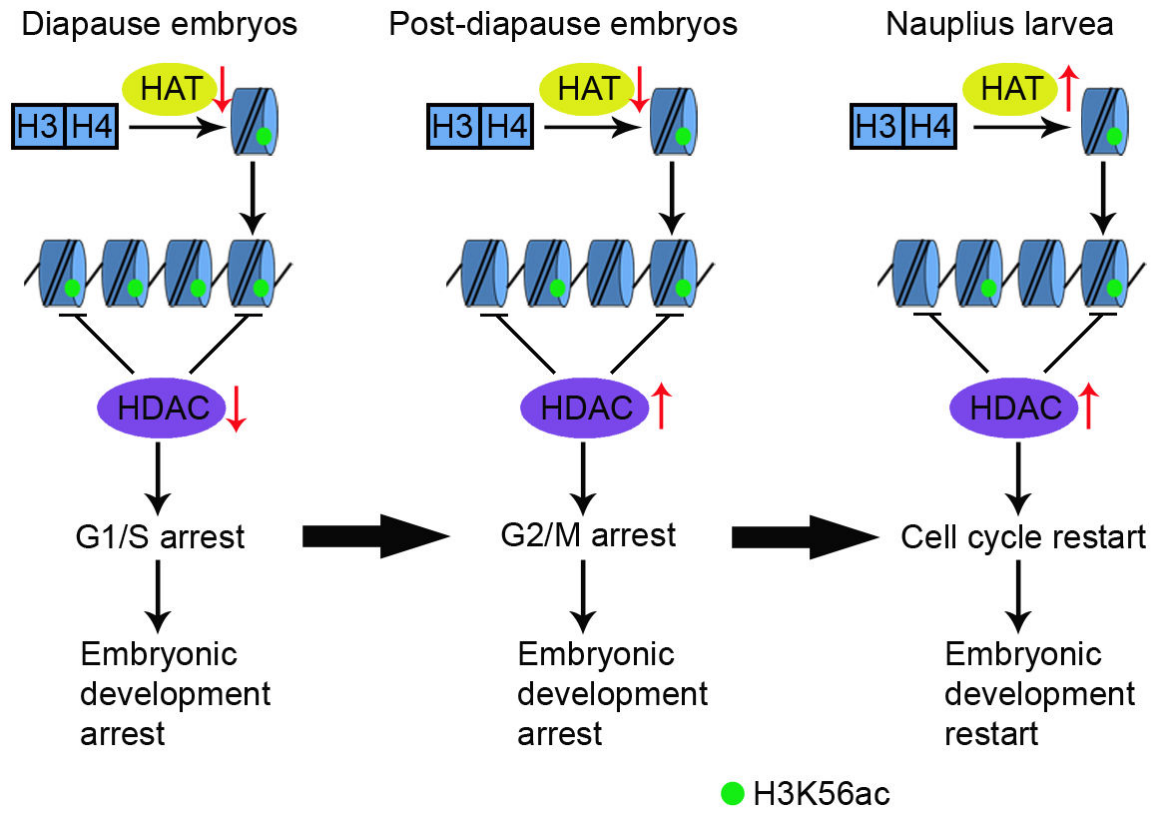
A working model for the regulation of *Artemia* diapause by H3K56ac. Green circles represent H3K56ac.

Taken together, we proposed a model to explain the relationship between the dynamic change of H3K56ac level and *Artemia* cell cycle and development (Figure 7). In diapause embryos, the repressed activities of HDACs and HATs resulted in the accumulation of H3K56ac on chromatin and low level of H3K56ac in the non-chromatin fraction, mediating cell cycle arrest at the G1/S checkpoint and the arrest of embryonic development at the gastrulae stage. After diapause termination, the level of H3K56ac on chromatin decreased, probably due to the restoration of HDAC activity. However, HAT activity in post-diapause embryos was still repressed, meaning the arrest of cell cycle transits from G1/S to G2/M phase. The activities of HATs and HDACs are both restored in nauplius larvae, meaning that the cell cycle and embryonic development can resume. Our results illustrate the influence of epigenetic regulation on diapause formation and termination in *Artemia*. Thus, our findings provide insight into the regulation of cell division during arrest of Artemia embryonic development and provide further insight into the functions of H3K56ac.

## Supporting Information

Figure S1 Alignments of amino acid sequences with other known Rtt109s and Asf1s. (**A**) represents the Rtt109 ortholog in *Artemia*. GenBank accession numbers of the sequences used are as follows: *Aedes aegypti*, EJY57367.1; *Daphnia* *pulex* , EFX66192.1; *Drosophila melanogaster*, AAB53050.1; *Homo sapiens*, NP_001420.2; *Xenopus laevis*, NP_001088637.1. (**B**) represents the ASF1 ortholog in *Artemia*. GenBank accession numbers of the sequences used are as follows: *Aedes aegypti*, XP_001656285.1; *Daphnia* *pulex* , EFX73971.1; *Drosophila melanogaster*, NP_524163.1; *Homo sapiens*, NP_054753.1; *Xenopus laevis*, NP_001080310.1; *Saccharomyces cerevisiae*, NP_012420.1.
(TIF)

Figure S2 Flow cytometry analysis of HeLa cells after different treatments. (TIF)
