# Supplementary figures and images for: Acetylation of Chromatin-Associated Histone H3 Lysine 56 Inhibits the Development of Encysted Artemia Embryos
Source: PLoS One. 2013 Jun 19;8(6):e68374. doi: 10.1371/journal.pone.0068374 (PMC3686719; doi:10.1371/journal.pone.0068374)

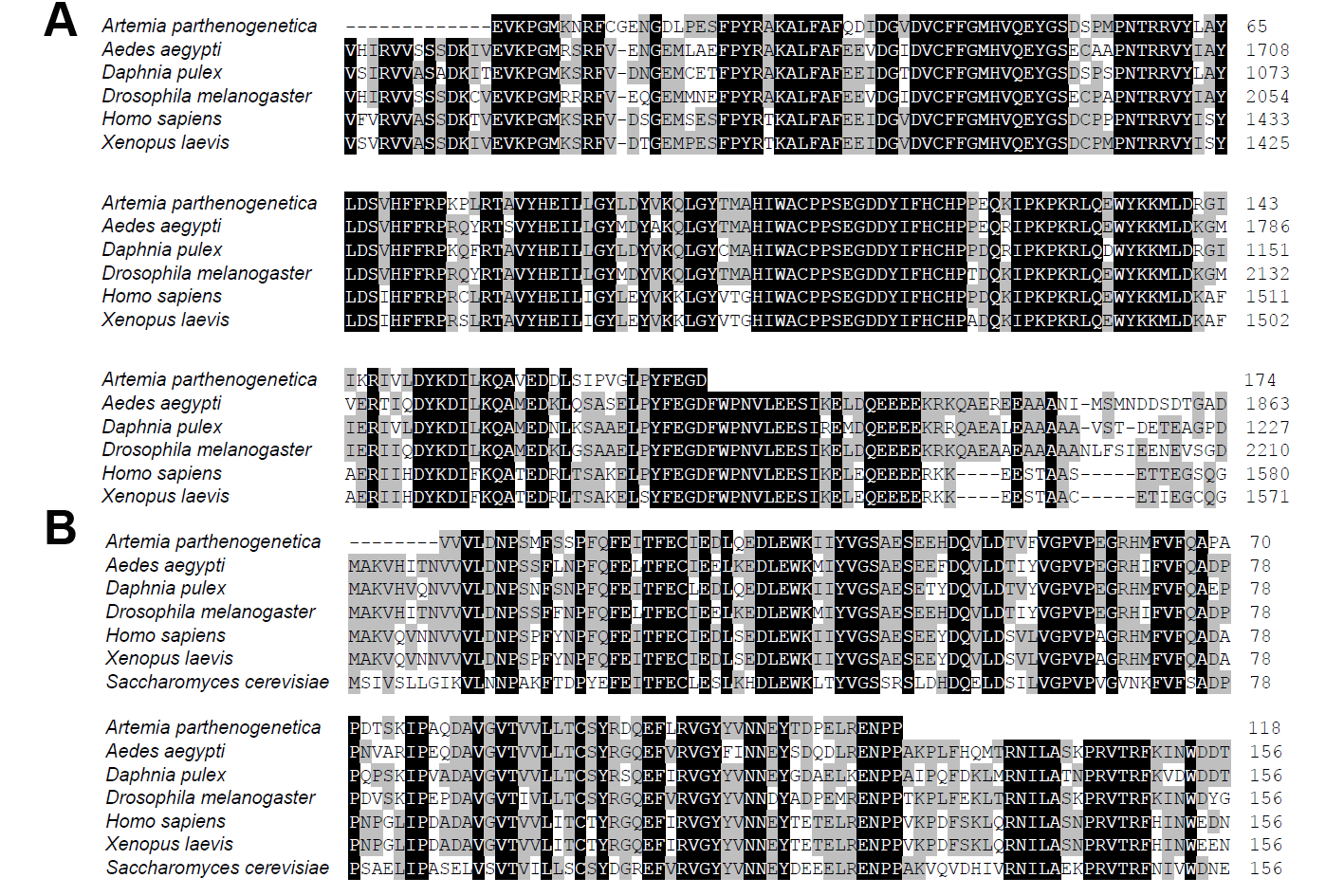

Supplement: Figure S1 — (A) represents the Rtt109 ortholog in Artemia. GenBank accession numbers of the sequences used are as follows: Aedes aegypti, EJY57367.1; Daphnia pulex , EFX66192.1; Drosophila melanogaster, AAB53050.1; Homo sapiens, NP_001420.2; Xenopus laevis, NP_001088637.1. (B) represents the ASF1 ortholog in Artemia. GenBank accession numbers of the sequences used are as follows: Aedes aegypti, XP_001656285.1; Daphnia pulex , EFX73971.1; Drosophila melanogaster, NP_524163.1; Homo sapiens, NP_054753.1; Xenopus laevis, NP_001080310.1; Saccharomyces cerevisiae, NP_012420.1. (TIF) [file pone.0068374.s001.tif]

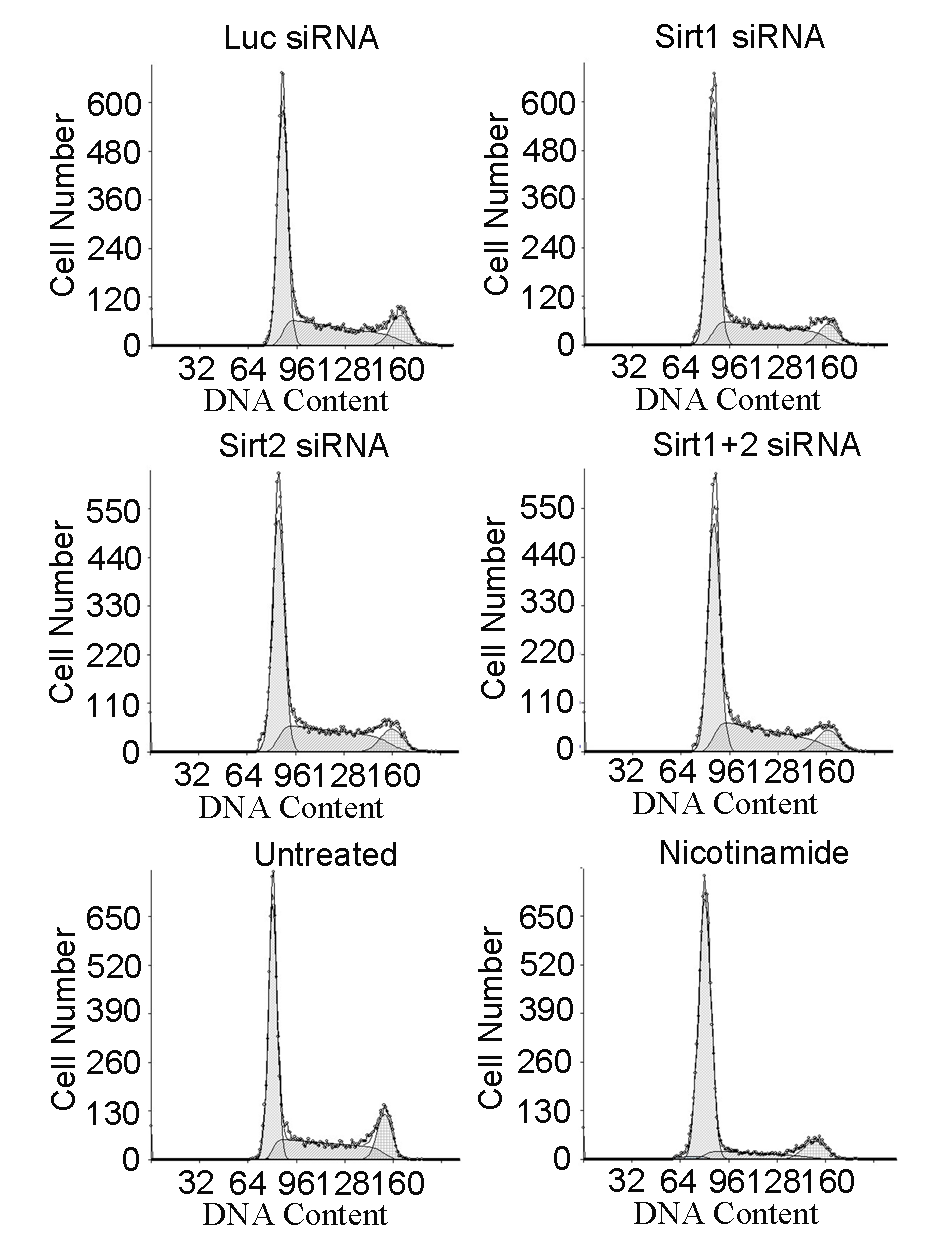

Supplement: Figure S2 — (TIF) [file pone.0068374.s002.tif]
